# Cell wall staining with Trypan blue enables quantitative analysis of morphological changes in yeast cells

**DOI:** 10.3389/fmicb.2015.00107

**Published:** 2015-02-11

**Authors:** Johannes Liesche, Magdalena Marek, Thomas Günther-Pomorski

**Affiliations:** Department of Plant and Environmental Sciences, University of CopenhagenCopenhagen, Denmark

**Keywords:** cell wall staining, Trypan blue, *Saccharomyces cerevisiae*, Calcofluor white, cell volume, Anaerobiosis, super-resolution microscopy, 3D structured illumination

## Abstract

Yeast cells are protected by a cell wall that plays an important role in the exchange of substances with the environment. The cell wall structure is dynamic and can adapt to different physiological states or environmental conditions. For the investigation of morphological changes, selective staining with fluorescent dyes is a valuable tool. Furthermore, cell wall staining is used to facilitate sub-cellular localization experiments with fluorescently-labeled proteins and the detection of yeast cells in non-fungal host tissues. Here, we report staining of *Saccharomyces cerevisiae* cell wall with Trypan Blue, which emits strong red fluorescence upon binding to chitin and yeast glucan; thereby, it facilitates cell wall analysis by confocal and super-resolution microscopy. The staining pattern of Trypan Blue was similar to that of the widely used UV-excitable, blue fluorescent cell wall stain Calcofluor White. Trypan Blue staining facilitated quantification of cell size and cell wall volume when utilizing the optical sectioning capacity of a confocal microscope. This enabled the quantification of morphological changes during growth under anaerobic conditions and in the presence of chemicals, demonstrating the potential of this approach for morphological investigations or screening assays.

## Introduction

Fungi are surrounded by a cell wall that is essential for maintenance of the cell shape and for regulation of the uptake of substances from the environment. The cell wall structure is dynamic and can adapt to different physiological states or environmental conditions. In yeast, major aspects, like cell wall composition and synthesis have been elucidated [see reviews by Bowman and Free (2006); Orlean (2012)], but important questions remain to be solved. For example, knowledge of the function of many wall-active proteins and the regulation of chitin and β 1-3 glucan synthases is incomplete, which prevents a more detailed understanding of changes in yeast cell wall composition in response to growth conditions (Aguilar-Uscanga and Francois, 2003; Kuznetsov et al., 2013). In addition, the cell wall serves as target for anti-fungal drugs (Gozalbo et al., 2004; Bowman and Free, 2006; Sun and Tong, 2014).

While selective staining of the cell wall with fluorescent dyes is routinely used in morphological and developmental studies, its potential in facilitating physiological investigations is has not been realized. One reason is that the prevalent cell wall dye, Calcofluor White (CFW, synonym Optical Brightener), fades relatively fast and requires a non-standard laser line for use in combination with confocal microscopy (Hoch et al., 2005). A possible alternative is Trypan Blue (TB), a dye commonly used for yeast cell viability assays (Karpova et al., 1993; Andrews and Stark, 2000). TB has also been used for visualization of the fungi groups Colletotrichum (Bhadauria et al., 2010) and Glomeromycota (Kumar et al., 2008). These studies showed that TB stains the cell walls of fungal cells but not of plant cells. Earlier, TB was used in bright field light microscopic staining of arbuscular mycorrhiza in a method that requires tissue clearing with KOH and acetic acid (Phillips and Hayman, 1970; Vierheilig and Piché, 1998). Vierheilig et al. (2001) hypothesize that TB binds to chitin, but no data on binding specificity is available so far. In addition, TB's spectral properties have not been determined.

Here, we report selective fluorescence staining of *Saccharomyces cerevisiae* cell wall with TB, characterize its spectral properties and, by using confocal microscopy, demonstrate its potential for quantitative analysis of changes in cell wall morphology, as exemplified for growth under anaerobic conditions and in CFW-containing medium. Growth under anaerobic conditions is known to affect the yeast cell and, specifically, the cell wall morphology (Aguilar-Uscanga and Francois, 2003). So far, only chemical and enzymatic methods have been used to investigate the changes caused by growth in the absence of oxygen. Likewise, CFW and the related compound Congo red are known to cause cell wall deformations at high concentrations (Vannini et al., 1983; Roncero and Durán, 1985), a property used to identify cell wall mutants (de Groot et al., 2001; Ram and Klis, 2006) and to test the involvement of proteins in cell wall synthesis (Kuznetsov et al., 2013). By confocal and super-resolution microscopy we show here that TB staining can be used to visualize fungal cell walls and to analyze differences in cell size and cell wall volume.

## Materials and methods

### Strains and growth conditions

The *S. cerevisiae* wild-type strain W303-1α (MATα *ade2-1 his3-11,15 leu2-3,112 trp1-1 ura3-1 can1-100)* was used in all experiments. Overnight cultures were grown at 28°C in YPD medium (1% w/v yeast extract, 2% w/v peptone, and 2% w/v glucose) to a density of OD_600_ = 0.8. For growth on plates, 2% agar was added to get solid media. Anaerobic conditions were generated by using an Anaerocult A system (Merck Milipore) and controlled by including an Anaerotest indicator stripe (Merck-Millipore) with the culture vial. When indicated, CFW (MP Biomedicals) and TB (Merck Millipore, “for microscopy,” order number: 111732) were added to the growth medium at a final concentration of 0.1 mg ml^−1^. This relatively high concentration was used in the context of the investigation of the dye's effect on cell morphology but not for staining (see below). Viability tests were performed with aliquots of the growth cultures using prodium iodide staining (Sigma-Aldrich, final concentration 1 μg ml^−1^).

### Staining procedure

Yeast cells were collected by centrifugation (900 g, 2 min, 20°C) and suspended in 200–300 μl phosphate buffered saline (PBS; 137 mM NaCl, 2.7 mM KCl, 10 mM Na_2_HPO_4_, 1.8 mM KH_2_PO_4_, pH 7.4) to OD_600_ = 1. TB was added to the cells in the tube or directly on the slide at a final concentration of 10 μg ml^−1^. Staining can be considered immediate, i.e., no incubation time is required. Additional washing steps after staining, including the resuspension in PBS and centrifugation repeated up to 3 times, were found to have minimal effect on the level of background fluorescence. Therefore, washing of cells after staining was generally omitted. Staining with CFW was performed similarly using a final concentration of 100 μg ml^−1^. Pontamine Fast Scarlet 4BS (Aldrich Rare Chemicals Library) was added to cell cultures 20 min before harvesting at concentrations up to 1 mg ml^−1^. Propidium iodide, used for vitality tests, was directly added to cells on the slide to a final concentration of 1 μg ml^−1^. Before imaging, cells were allowed to settle for 1 min before applying the cover slip. Standard microscopy glass slides and cover slips were used.

For the analysis of staining specificity, commercially available extracts of various cell wall components were tested. Powders, specified as high purity by the supplier, of chitin from shrimp shells (Sigma, Product number C9752), glucan “from baker's yeast” (Sigma, G5011), xyloglucan from tamarind (Megazyme, 95% purity, P-XYGLN), beta glucans from barley (Megazyme, 95% purity, P-BGBM), esterified pectin from citrus (Sigma, 85% esterified, P9561), CM-cellulose (Megazyme, S-ACMC), Polygalacturonic acid (Megazyme, >95% purity, P-PGACT) and xylan from beechwood (Sigma, >90% xylose residues, X4252) were used. The components were dissolved at a concentration of 0.5% (w/v) in water or 5N NaOH in case of chitin and the glucans. Chitin required 30 min incubation at 98°C to dissolve.

To test the fluorescence staining of the different cell wall components in powder form several microgram of powder were deposited on a slide and stained by adding 15 μl of 10 μg ml^−1^ TB in PBS and a coverslip before analysis on a confocal microscope.

### Spectroscopic analysis

Absorption was measured on a Genesys 10 Bio (Thermo Scientific) spectrophotometer, while fluorescence emission intensity was determined using a Fluoromax-4 (Horiba Jobin Yvon). First, unstained samples of the dissolved cell wall components were analyzed. Afterwards, TB was added to the samples to the final concentration of 10 μg ml^−1^ and analyzed. Absorption spectra were recorded from 400 to 700 nm. Fluorescence spectra were recorded from 640 to 750 nm using excitation at 620 nm. Pure water or NaOH served as a baseline reference. The values for unstained samples were subtracted from the values of TB-stained samples.

### Image acquisition

A confocal laser scanning microscope (Leica SP5-X, Leica Microsystems) equipped with a 63× (numerical aperture 1.2) water-immersion objective was used for all microscopic studies, except super-resolution imaging. A freely tunable white light laser was used for excitation. Unless stated otherwise, the following settings were used: TB, 620 nm excitation, 627–720 nm detection; CFW, 355 nm excitation, 400–460 nm detection; Pontamine Fast Scarlet, 500 nm excitation, 580–650 nm detection; Propidium iodide, 535 nm excitation, 590–660 nm detection. The excitation-emission scans (λ^2^-scans) were conducted with the following parameters: 470–650 nm excitation range, 19 steps with 10 nm step size, constant laser power, 480–720 nm detection range, 20 nm detection band width, 12 steps with 20 nm step size. The staining intensity of cell wall components was measured by acquiring an image of the powder surface using the exactly same settings for all measurements.

Super-resolution microscopy was performed using a Zeiss Elyra PS1 instrument. TB was excited by a 642 nm laser and emitted light was filtered with a 650 nm long-pass filter. Light was detected on a sCMOS camera (PCO Edge 4.2). A 100× 1.46 NA Zeiss Apochromat oil-immersion objective was used together with a 51 μm grating. Z-stacks were recorded with 3 phase-changes and 3 grating rotations for each section.

### Image processing

Results from the λ^2^-scans were visualized in Leica LAS software (Leica Microsystems). The staining intensity of different cell wall components was determined by drawing a region of interest around the stained area and measuring the mean gray value using ImageJ. The background signal, which was the same in all cases, was subtracted. Average gray values and standard deviation were determined from 5 repetitions.

Cell wall volume and total cell volume were determined in Volocity (Version 6.3, PerkinElmer). An object detection algorithm with the following components was used: “Find objects using % intensity” with the lower boundary at 3%, “Clip objects to region of interest,” “remove noise from objects” (fine filter), “exclude objects by size” with minimum size of 20 μm^3^. For measurement of cell size, a “close” command was added with 10 iterations. Regions of interest were drawn around single cells using a Cintiq 21ux interactive pen display (Wacom), as this was found to be more efficient for restricting each object to a single cell compared to a software command, specifically when cells were touching. In case of budding cells, the mother cell and the bud were treated as two separate cells.

The image processing of super-resolution images was performed using Zeiss ZEN software (Version 2013). The structured illumination wizard was used with the following settings: SR frequency weighing 1, Baseline cut, theoretical point spread function and automatic noise filtering. Afterwards, images were cropped and the dynamic range adjusted.

### Statistical analysis

Results are given as mean ± S.D. Statistical analysis was done using Student's *t*-test. A value of *P* < 0.05 was considered as statistically significant. All experiments were repeated at least three times (separate cell preparations) if not stated otherwise.

## Results

### Trypan blue stains chitin and glucans and its spectral properties are suited for confocal microscopy

**Figure 1:**
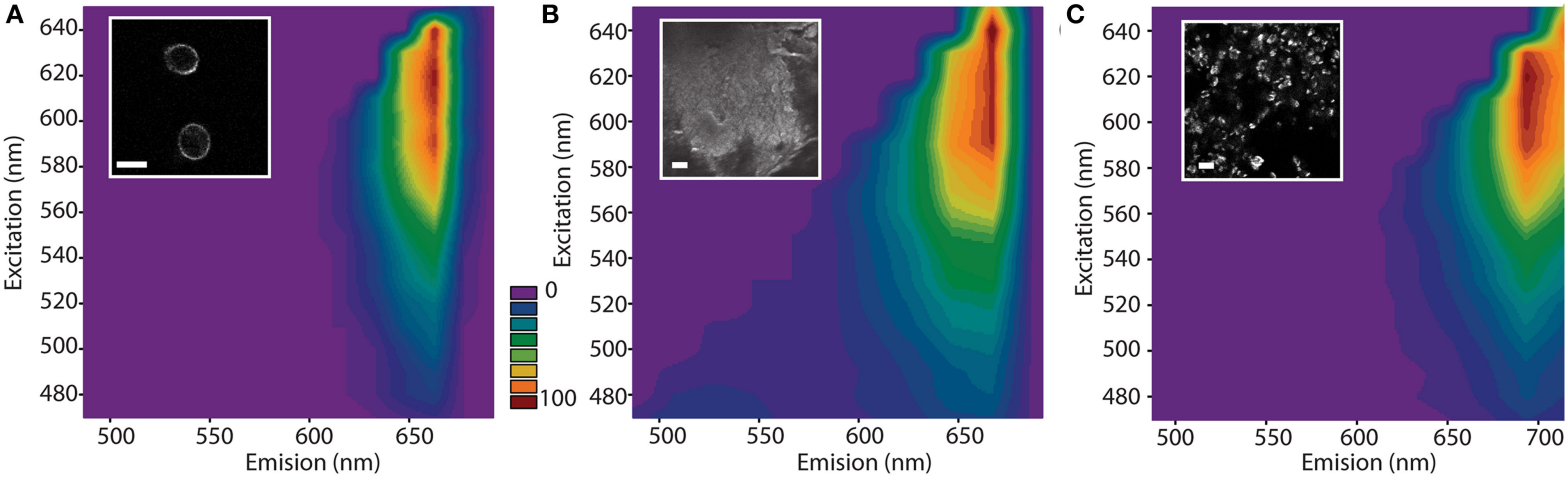
**Excitation-emission scan and exemplary images of Trypan Blue-stained yeast cells (A), chitin (B), and yeast glucans (C)**. The scan was performed on a confocal microscope with freely selectable excitation and emission settings. Scale bars, 5 μm. The color bar represents the normalized fluorescence intensity with respect to the maximum and minimum level.

Addition of TB to *S. cerevisiae* cells resulted in ring-like fluorescent staining of the cell wall (Figure 1A, insert). Analysis of the spectral properties of TB-stained cell walls using excitation-emission scans (λ^2^-scans) showed a broad excitation with peak at 580–640 nm (Figure 1A). This range matches with the 633 and 561 nm standard laser lines available on most confocal microscope setups. Emission was restricted to a narrow peak around 670 nm (Figure 1A). Cell autofluorescence did not interfere with TB fluorescence in the emission range of 520–590 nm (Supplementary Figure S1A). Compared to stained live cells, TB-stained dead cells showed excitation–emission maxima that were slightly shifted about 20–30 nm toward shorter wavelength (Supplementary Figure S1B).

The fluorescence spectrum of TB-stained live cells matched that of TB-stained pure chitin (Figure 1B), indicating that TB fluoresces upon binding to chitin. In addition, glucans extracted from yeast cell walls are stained with a similar excitation-emission pattern, although the emission peak is broader and shifted about 30 nm to longer wavelength (Figure 1C).

**Figure 2:**
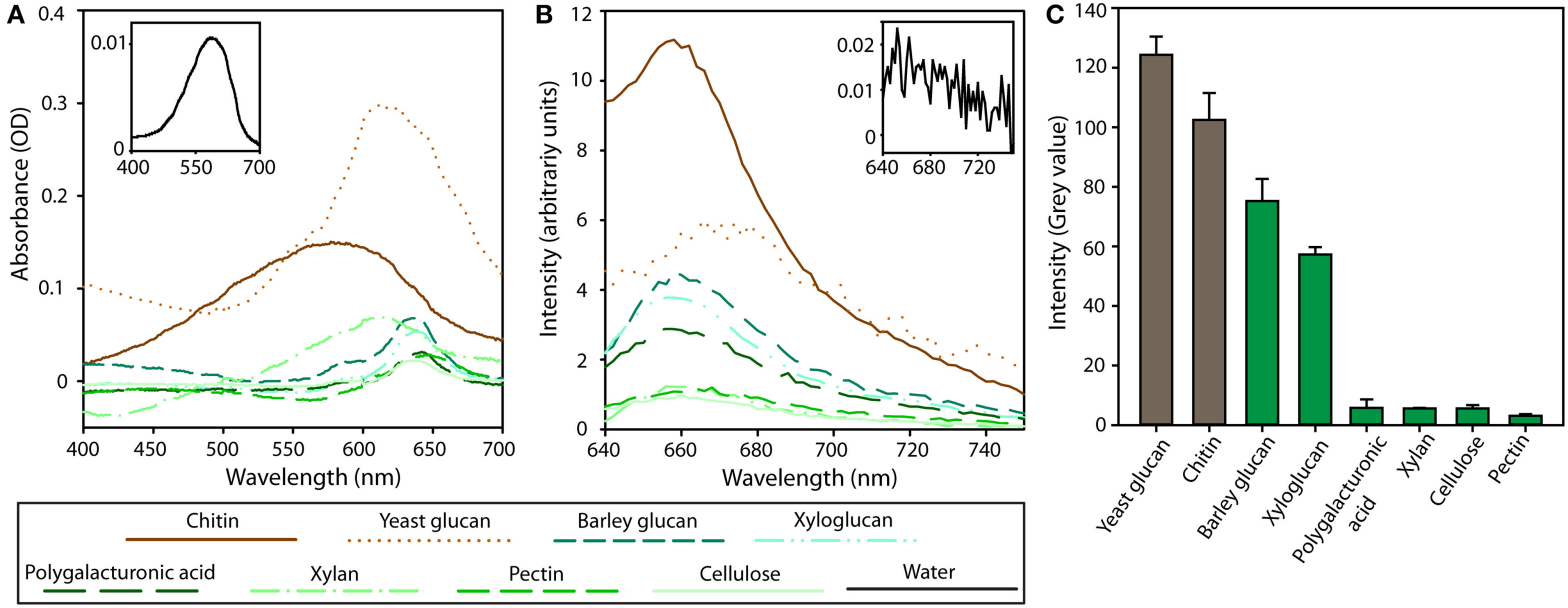
**Absorption (A) and fluorescence (B, C) of cell wall components of fungi (brown lines/bars) and plants (green lines/bars) after Trypan Blue staining. (A,B)** Cell wall components were dissolved at a concentration of 0.5% (w/v) in water or NaOH containing 10 μg ml^−1^ Trypan Blue and absorbance **(A)** and fluorescence **(B)** spectra were recorded. Inserts show spectra for Trypan Blue in aqueous solution. Fluorescence spectra were recorded with excitation at 620 nm. Control values from unstained samples were subtracted. In **(C)** purified powder of the indicated cell wall components was stained with 10 μg ml^−1^ Trypan Blue and imaged on a confocal microscope with the excitation wavelength of 620 nm. Mean fluorescence intensities of the in-focus surfaces are presented. Error bars represent standard deviation from 5 measurements. The differences between visibly stained components (gray value above 50) are all statistically significant (*p* < 0.01), while the difference between minimally stained components (gray value below 20) are not significant. Note that quantitative differences of fluorescence intensity values between spectrophotometric **(B)** and microscopic **(C)** analysis could be due to the different form (dissolved vs. powder) or conditions (neutral vs. acidic).

Similarly, the analysis of absorbance and fluorescence staining intensities of a wide range of cell wall components showed that TB stains chitin and yeast glucans strongest of all tested cell wall components (Figure 2). Spectrophotometric bulk measurements revealed a strong increase in TB absorbance in presence of yeast glucan and chitin as compared to plant cell wall components (Figure 2A). Moreover, in presence of chitin a distinct blue-shift of the TB absorption peak was observed (from 610 nm of free TB to 574 nm of chitin-bound TB) (Figure 2A). This blue shift of 36 nm indicates metachromatic interaction between the dye and polymer and, thereby, a specific mode of interaction between TB and chitin. Despite the relatively low absorption, TB-stained chitin displayed the highest fluorescence emission intensity (Figure 2B). In contrast, the fluorescence of TB-stained yeast glucan was relatively low compared to the very high absorbance (Figure 2). Of the plant cell wall components, barley glucan and xyloglucan showed fluorescence at a level of about 35% of chitin (Figure 2B). The data was complemented by measurements on undissolved cell wall polymers in order to test whether dissolving, which could compromise the polymer's structures, influence the staining efficiency with TB. Dissolving can lead to alteration of the structure of cell wall polymers (Einbu et al., 2004). When TB was added to the powder, yeast glucan showed a slightly, but significantly, higher fluorescence intensity than chitin (Figure 2C). Glucans and xyloglucans extracted from barley and tamarind cell walls, respectively, were also stained, reaching 61% and 46% of the yeast glucan level, respectively. Other components from plant cell walls showed signal intensity slightly above background levels (Figure 2C). In addition, it was tested whether TB can stain lipids was tested. Addition of TB to lipid vesicles did not result in any detectable fluorescence signal (Supplementary Figure S2).

### Trypan blue staining shows the same patterns as calcofluor white staining but the signal is more stable

**Figure 3:**
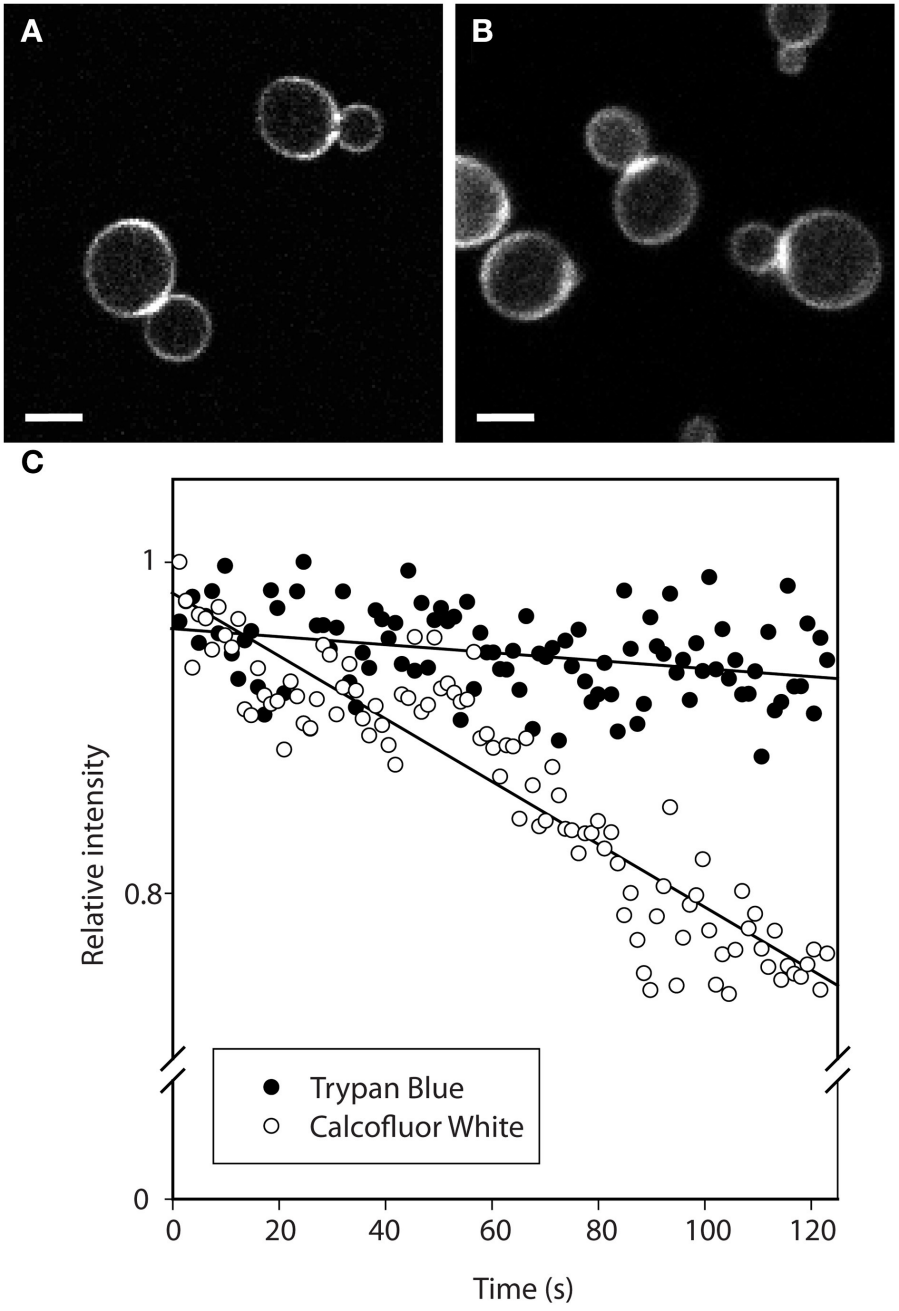
**Comparison of cell wall staining with Trypan Blue (A) and Calcofluor White (B) and their respective signal bleaching (C)**. Stained cells were scanned repeatedly on a confocal microscope and the signal intensity was normalized to the signal at time zero. The connecting lines were obtained by linear regression. Scale bars in **(A,B)**, 5 μm.

TB staining of *S. cerevisiae* cell walls was compared to Pontamine Fast Scarlet and the commonly used CFW. The fluorescence staining pattern of TB-stained cells matched that of cells stained with CFW (Figures 3A,B). The typical ring staining was visible and the signal was stronger at the cell wall region between mother and daughter cells, indicating chitin accumulation at the septum. In contrast to a previous report (Hoch et al., 2005), Pontamine Fast Scarlet staining did not result in any fluorescence signal, even at high concentration and after long incubation times (data not shown).

The TB signal was found to be very stable. After 2 min, signal intensity decreased by about 5% (Figure 3C). This compares favorably with CW signal that, within 2 min, decreased by 25%. Even after 600 frames, corresponding to about 15 min of continuous scanning, the signal from TB-stained cell walls remained within 85% of original intensity. Thus, TB enables long-term observations.

### Trypan blue staining enables quantification of wall volume and total cell volume

Next, we applied TB staining to quantify cell wall and total cell volume of individual yeast cells. Classical analysis is based on calculating the volume of a sphere using the cell diameter in one xy-image. However, the measured cell diameter does often not correspond to the maximum diameter of the cell. Additional inaccuracy is introduced through cell shapes diverging from a sphere.

**Figure 4:**
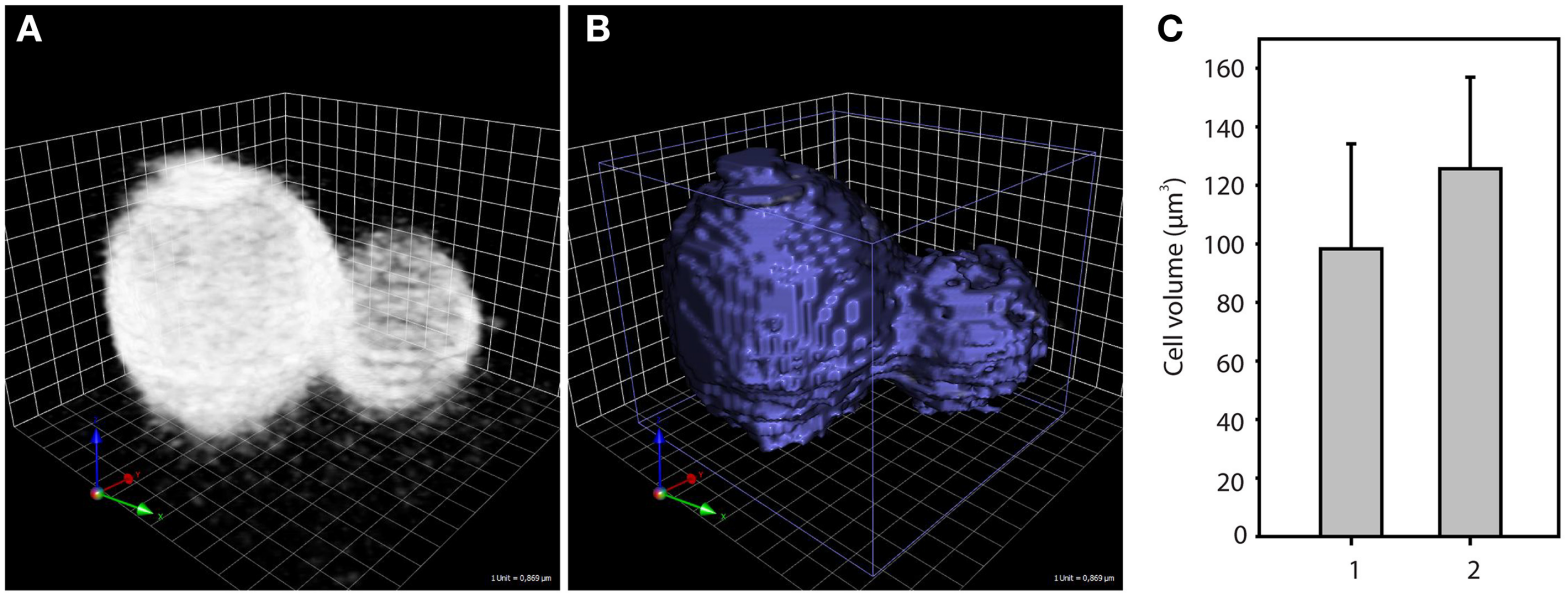
**Trypan Blue staining enables cell and cell wall volume quantification**. Image stacks of Trypan Blue stained yeast cells acquired by confocal microscopy were rendered in 3D **(A)**. Based on the fluorescence signal, an object corresponding to the cell wall was defined, which is displayed here in 3D surface rendering mode **(B)**. Comparison of cell volume of 35 cells **(C)** determined based on cell diameters in a single xy-image (1) or provided by 3D object detection based on the whole xyz-image stack (2); In **(A, B)**, 1 unit corresponds to 0.8 μm.

By acquiring image stacks according to the Nyquist criterion (van der Voort and Strasters, 1995), which guarantees that even the smallest resolvable structure is adequately sampled, image information is collected that can be rendered in 3D (Figure 4A). Using an object detection algorithm, a volume corresponding to the fluorescence signal can be defined (Figure 4B). The volume that is determined in this way corresponds to the cell wall volume. The fluorescence signal domain is larger than the cell wall domain, which falls below the resolution limit of conventional fluorescence microscopes (wall thickness about 30 nm, resolution limit about 200 nm). An object corresponding to the whole cell was created by adding an additional closing command to the algorithm. Comparing cell volume measurements with this method to the classical analysis based on calculating the volume of a sphere using the cell diameter in one xy-image, the obvious underestimation of the classical method becomes apparent (Figure 4C). In conclusion, cell wall staining with TB in combination with optical sectioning using confocal microscopy enables quantification of cell wall and cell volume.

### Trypan blue staining shows changes in cell size and wall volume of cells grown anaerobically grown or at high calcofluor white concentrations

**Figure 5:**
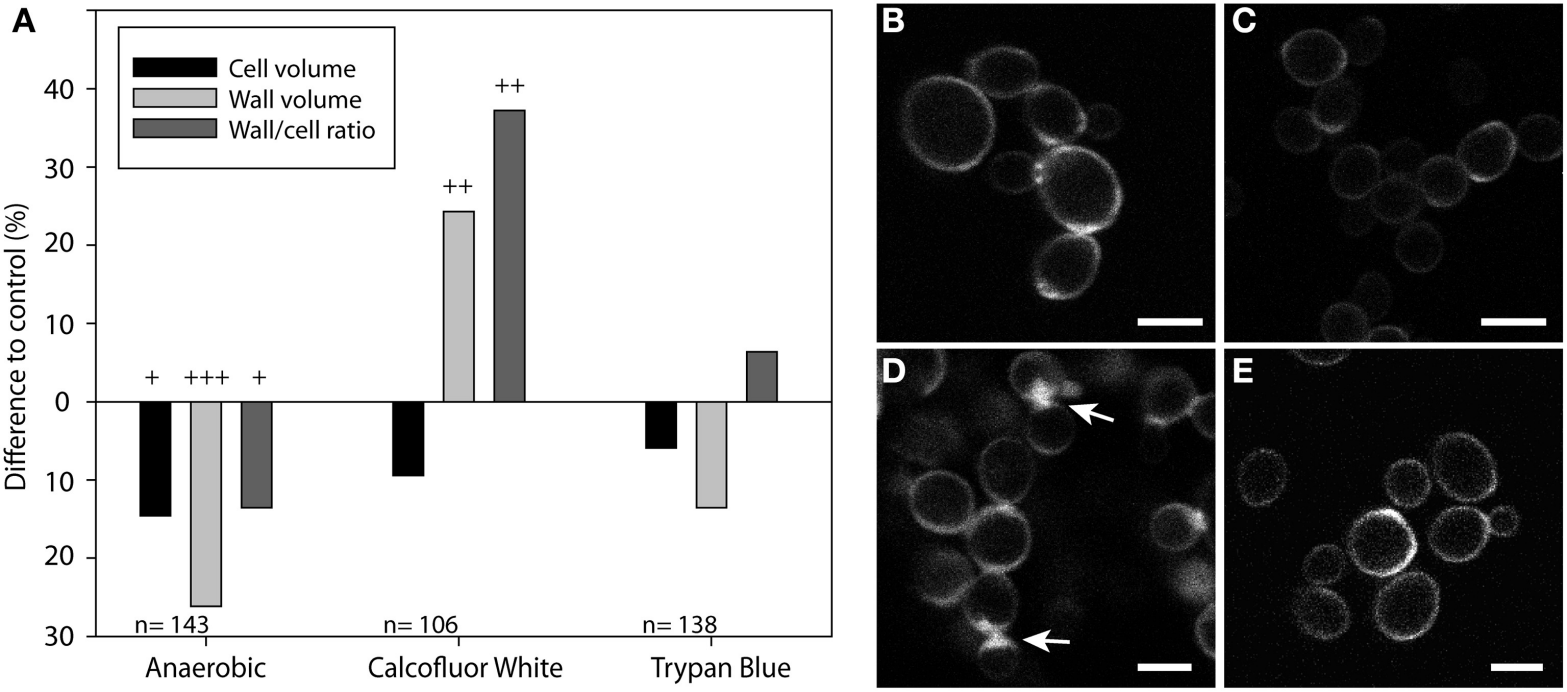
**Effect of growth conditions on cell size and wall volume analyzed by Trypan Blue staining**. *S. cerevisiae* cells grown overnight under anaerobic conditions or in medium containing high concentrations (0.1 mg ml^−1^) of Calcofluor White (CFW) or Trypan Blue (TB) were stained with TB and analyzed by confocal fluorescence microscopy. Based on image 3D-renderning (cf. Figure 4), cell volume, wall volume, and cell/wall volume ratio were determined for the indicated number (n) of cells and expressed as percentage relative to untreated, aerobically grown cells (control cell volume, 123.6 μm^3^; control wall volume, 66 μm^3^; control wall/cell volume ratio, 0.53) **(A)**. Representative images of TB-stained cells grown under control **(B)**, anaerobic conditions **(C)**, with Calcofluor White **(D)** or Trypan Blue **(E)** are shown. Cell wall ingrowth caused by Calcofluor White **(D)** are marked by arrows. Dead cells were excluded from the analysis based on an intracellular staining by TB. These cells accounted for about 4% of CFW-incubated cells and about 0.5% in all other cultures. Statistical significance is indicated in **(A)**: *p* < 0.001 (+++), *p* < 0.01 (++), *p* < 0.05 (+). Scale bars, 5 μm.

Wall and cell volume analysis based on TB staining was then used to assess morphological changes in *S. cerevisiae* cells grown overnight under anaerobic conditions or in medium containing high concentrations (0.1 mg ml^−1^) of CFW or TB. Based on image 3D-renderning (cf. Figure 4), cell volume, wall volume and cell/wall volume ratio were determined and expressed relative to untreated, aerobically grown cells (Figure 5). Cells grown anaerobically were generally smaller than control cells grown under aerobic conditions (Figure 5A). Interestingly, the cell wall volume was significantly smaller, even when corrected for the smaller cell size. Contrastingly, the cell wall volume of cells grown in the presence of CFW was increased by more than 30% compared to the control cells grown without CFW, while cell size did not differ significantly (Figure 5A). No significant changes in wall volume or cell size were observed for cell grown in the presence of TB at 10 times higher concentration than used for staining (Figure 5A).

Consistent with these results analysis of fluorescent images of showed that the cell wall of anaerobically grown cells was stained less intense by TB as compared with control cells (cf. Figures 5B,C), while strongly stained areas with aberrant structures were visible in the walls of cells grown in the presence of CFW (Figure 5D). Cells grown in the presence of TB displayed a staining similar to that of control cells (cf Figures 5B,E).

**Figure 6:**
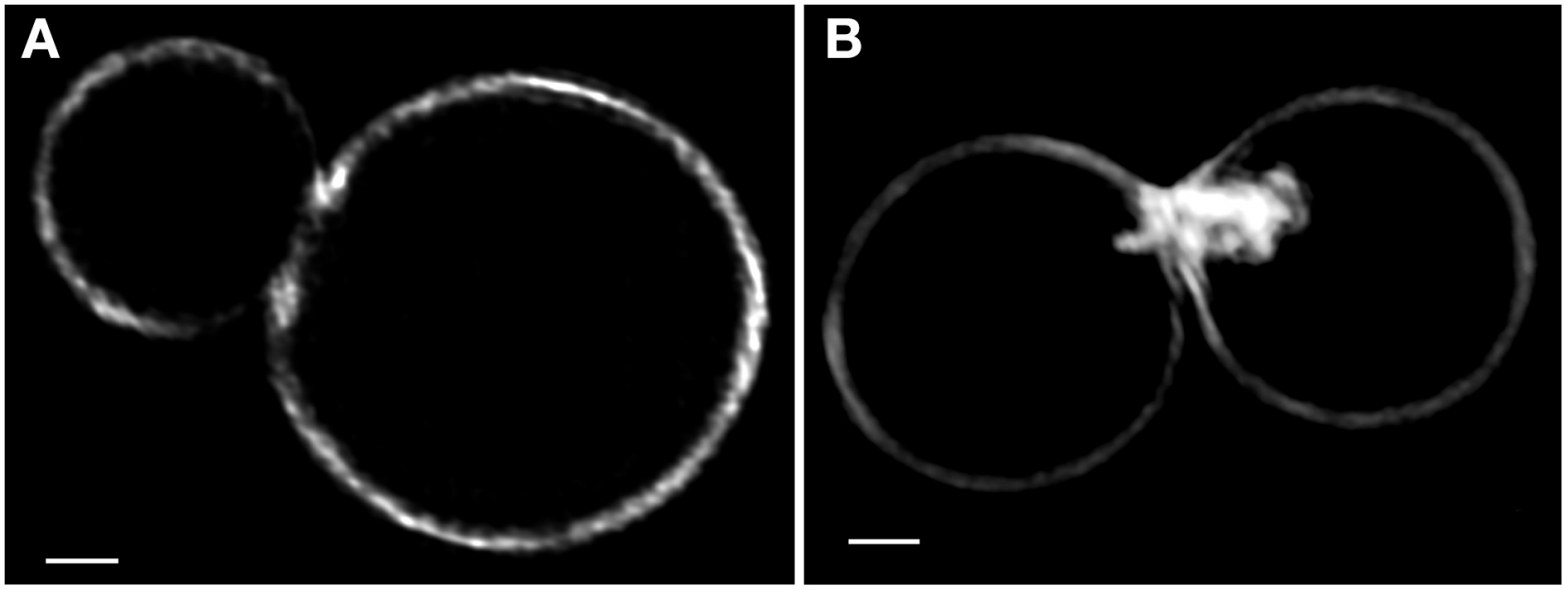
**Super-resolution images of Trypan Blue stained yeast cells**. Cells were grown aerobically overnight in the absence **(A)** and presence **(B)** of 0.1 mg ml^−1^ Calcofluor White, then stained with Trypan Blue and visualized by the super-resolution microscopy technique 3D structured illumination. Scale bars, 1 μm.

Furthermore, the aberrant cell wall morphology caused by growth in the presence of high concentrations of CFW was visualized in detail using the super-resolution microscopy technique 3D structured illumination (Figure 6). In some of the cells grown in the presence of CFW, wall ingrowth can be seen, specifically at the budding sites (Figure 6B). Such ingrowth was never observed in cells grown under control conditions (Figure 6A).

## Discussion

Live-cell stains of cell walls are an important tool for the morphological and developmental analysis of yeast cells. TB has not been reported as a cell wall stain for yeast cells before. In this study, we demonstrated that its spectral properties are superior to the conventional cell wall fluorophore CFW. The excitation and emission characteristics of TB are well suited for standard confocal microscope setups. Whereas CFW visualization requires a UV laser, reasonable excitation of TB is achieved at the standard laser wavelength of 561 nm or 633 nm. Staining is immediate, with virtually no background autofluorescence and high signal stability. TB is widely used as a live-dead marker in *S. cerevisiae* (see for example Kucsera et al., 2000; Lalonde et al., 2014) and other eukaryotic cells. In line with previous studies (Harrison et al., 1981; Avelar-Freitas et al., 2014), we observed that TB-stained dead cells emit fluorescence at about 650 nm. Since these fluorescence characteristics are similar to those of living cell wall-stained cells, living and dead cells cannot be simply discriminated based on the fluorescence signal. However, the subcellular signal pattern and the blue staining visible on bright field images enables convenient identification of dead cells in confocal microscopy.

It should be noted that TB is a quencher of green fluorescence (Sahlin et al., 1983). On one hand, this limits its application in studies where green-labeled proteins, lipids or other molecules at the cell surface need to be observed concomitantly with the stained cell wall. On the other hand, TB can be used as a non-permeating quencher of extracellularly accessible green fluorophores, for example to quantify internalization of labeled molecules (Ramarao and Meyer, 2001; van Bracht et al., 2014).

When TB was shown to stain the cell wall of mycorrhizal fungi, but not their plant hosts, it was suspected that fluorescence is caused by specific binding to chitin (Vierheilig et al., 2001). This assumption was partly corroborated here. First, we demonstrated that TB stains pure chitin with the fluorescence spectrum matching that of TB-stained *S. cerevisiae* cell walls. Second, we observed a shift in the absorption wavelength for TB in presence of chitin as compared to minor λ_max_ changes in presence of other plant cell wall components. Third, despite the relatively low absorption, TB-stained chitin displayed the highest fluorescence emission intensity. Conceivably, this specific interaction of TB with chitin could be the reason for the observed specificity for fungal cell walls. In addition, even though some components of plant cell walls, like glucans and xyloglucans, were also stained to a certain degree when tested *in vitro*, this might not lead to a clear signal *in vivo*, because of the relatively low abundance of these components in cell walls. In fungal cell walls, chitin and glucan make up the majority of wall dry weight, 50–60% in *S. cerevisiae* cell walls (Klis et al., 2002). In contrast, glucans and xyloglucans constitute a minor part of the plant cell wall, for example about 20% in *Arabidopsis thaliana* (Zablackis et al., 1995). Furthermore, the accessibility of glucans in the plant cell wall might be reduced by pectins that cover the space between cellulose and hemicellulose. Very abundant mannoproteins in the fungal cell walls are also likely to stain by TB and, thereby, contribute to a high staining intensity. Considering this specificity, TB can be a powerful tool for the analysis of yeast infection in plant as well as in mammalian systems, potentially facilitating the development of anti-fungal drugs.

We illustrated the potential of TB staining for morphological investigations on cells cultivated under anaerobic conditions and in the presence of high concentrations of CFW. Cultivation under anaerobic conditions is known to have profound multi-level effects, including growth rate reduction and modification of the cell wall composition (Aguilar-Uscanga and Francois, 2003). Also chemicals can affect cell morphology. Indeed, CFW was shown to alter the assembly of chitin microfibrils in yeasts (Durán and Cabib, 1978) and reduce chitin degradation *in vitro* by 50% (Roncero and Durán, 1985). Cells grown in the presence of CFW form more multicellular aggregates, putatively because cells were repeatedly budding without completing the separation between mother and daughter cells, which is supported by the observation of abnormally thick septa (Roncero and Durán, 1985). These effects were generally measured with destructive methods, specifically biochemical analysis of cell wall composition or transmission electron microscopy. Especially in the context of screening of mutant libraries, a fast and reliable method to detect morphological changes related to chitin in living cells could facilitate the experimental workflow. It was shown here that TB staining in combination with confocal microscopy and 3D image analysis can indeed detect changes in cell wall morphology. Both, the decrease in cell wall thickness in anaerobically grown cells and the additional chitin formation caused by presence of CFW in the media could be detected. Since TB does not have a significant effect on cell morphology itself, even at the relatively high concentration tested here, it could be used in efficient screens for cell wall mutants.

The progress in imaging technology over the last decade led to a considerable increase in resolution of light microscopes. Different methods have been developed to overcome the diffraction barrier, of which 3D structured illumination has the advantage of working with standard fluorophores and not being limited to a narrow z-range (Schermelleh et al., 2010). As such, it was used here to image TB-stained chitin at a resolution of about 100 nm. At this resolution, the structure of the aberrant wall ingrowth caused by CFW-containing medium could be visualized, demonstrating their similarity to cell wall ingrowth caused by Congo Red as seen by electron microscopy (Vannini et al., 1983). The ingrowth and their particular shape observed here are presumably the result of the dual effect of increased chitin synthesis and defective polymerization. The specific rate of chitin synthesis was shown to be increased by CFW in *S. cerevisiae* (Roncero and Durán, 1985; Bulawa et al., 1986). At the same time, CFW and Congo Red are known to inhibit the polymerization of chitin microfibrils and binding of chitin to β (1,3)-glucan fibers (Merzendorfer, 2013). The results also show that when CFW staining is used, care has to be taken that incubation time and concentration are kept at a low enough level not to affect cell morphology.

## Conflict of interest statement

The authors declare that the research was conducted in the absence of any commercial or financial relationships that could be construed as a potential conflict of interest.
