# Supplementary material for: Cell wall staining with Trypan blue enables quantitative analysis of morphological changes in yeast cells
Source: Front Microbiol. 2015 Feb 11;6:107. doi: 10.3389/fmicb.2015.00107 (PMC4324143; doi:10.3389/fmicb.2015.00107)
Supplement: Supplementary file 1 [file DataSheet1.PDF]

Supplementary Material

Cell wall staining with Trypan Blue enables quantitative analysis of morphological changes in yeast cells.

Johannes Liesche[1]*, Magdalena Marek[1], Thomas Günther Pomorski[1]

[1]Department of Plant and Environmental Sciences, University of Copenhagen, 1871 Frederiksberg C, Copenhagen, Denmark

*** Correspondence:** Johannes Liesche, Department of Plant and Environmental Sciences, University of Copenhagen, Thorvaldsensvej 40, 1871 Frederiksberg C, Copenhagen, Denmark.
joli@plen.ku.dk

1. Supplementary Figures and Tables

1.1. Suplementary Figures

Supplementary Figure 1. Excitation-emission scan and exemplary images of unstained yeast cells as a control for autofluorescence (A) and Trypan Blue-stained dead cells (B). The scan was performed on a confocal microscope with freely selectable excitation and emission settings. Note that the detector sensitivity for (A) is about two times higher than in (B) to detect the low autofluorescence signal, leading to a high noise level. Cells in (B) were killed by incubation at 98°C for 5 min. The color bar represents the normalized fluorescence intensity with respect to the maximum and minimum level.

Supplementary Figure 2. Trypan Blue does not stain lipids. Giant unilamellar vesicles were produced from 1,2-dioleoyl-sn-phosphatidylcholine lipids as described by Papdopulus et al. (2007). The vesicles, visible in differential interference contrast (A), do not show any fluorescence staining when incubated in 10 µg ml^{-1} Trypan Blue solution (B). Insert in (B) shows the line profile through one vesicle, validating that intensity levels do not increase above background. Imaging was performed on a Leica DMI6000 fluorescence microscope with adequate filter settings. Scale bars, 10 µm.

2. References for Supplementary Data

Papadopulos A, Vehring S, López-Montero I, Kutschenko L, Stöckl M, Devaux PF, Kozlov M, Pomorski T, Herrmann A. Flippase Activity Detected with Unlabeled Lipids by Shape Changes of Giant Unilamellar Vesicles. J Biol Chem 2007, 282: 15559-15568.
