# Supplementary figures and images for: Cell wall staining with Trypan blue enables quantitative analysis of morphological changes in yeast cells
Source: Front Microbiol. 2015 Feb 11;6:107. doi: 10.3389/fmicb.2015.00107 (PMC4324143; doi:10.3389/fmicb.2015.00107)

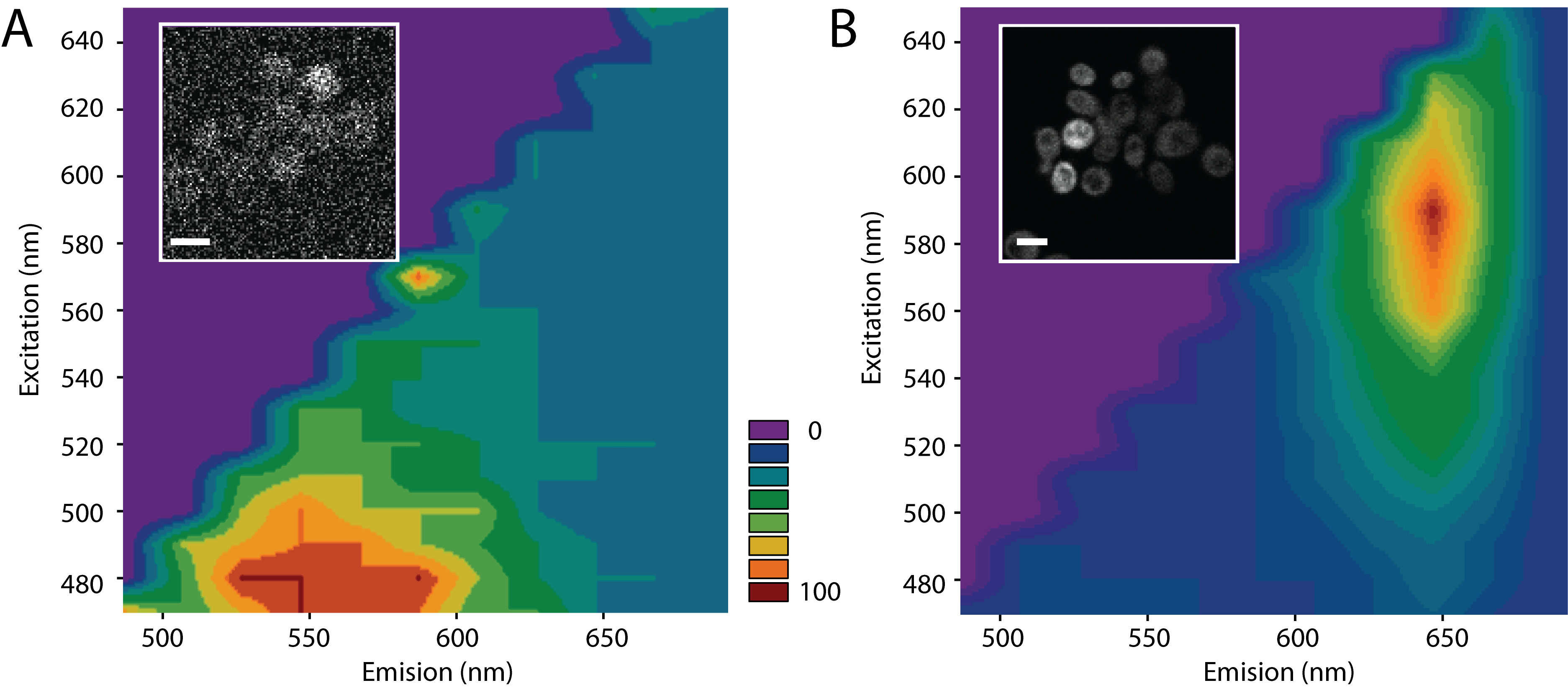

Supplement: Supplementary file 2 [file Image1.TIF]

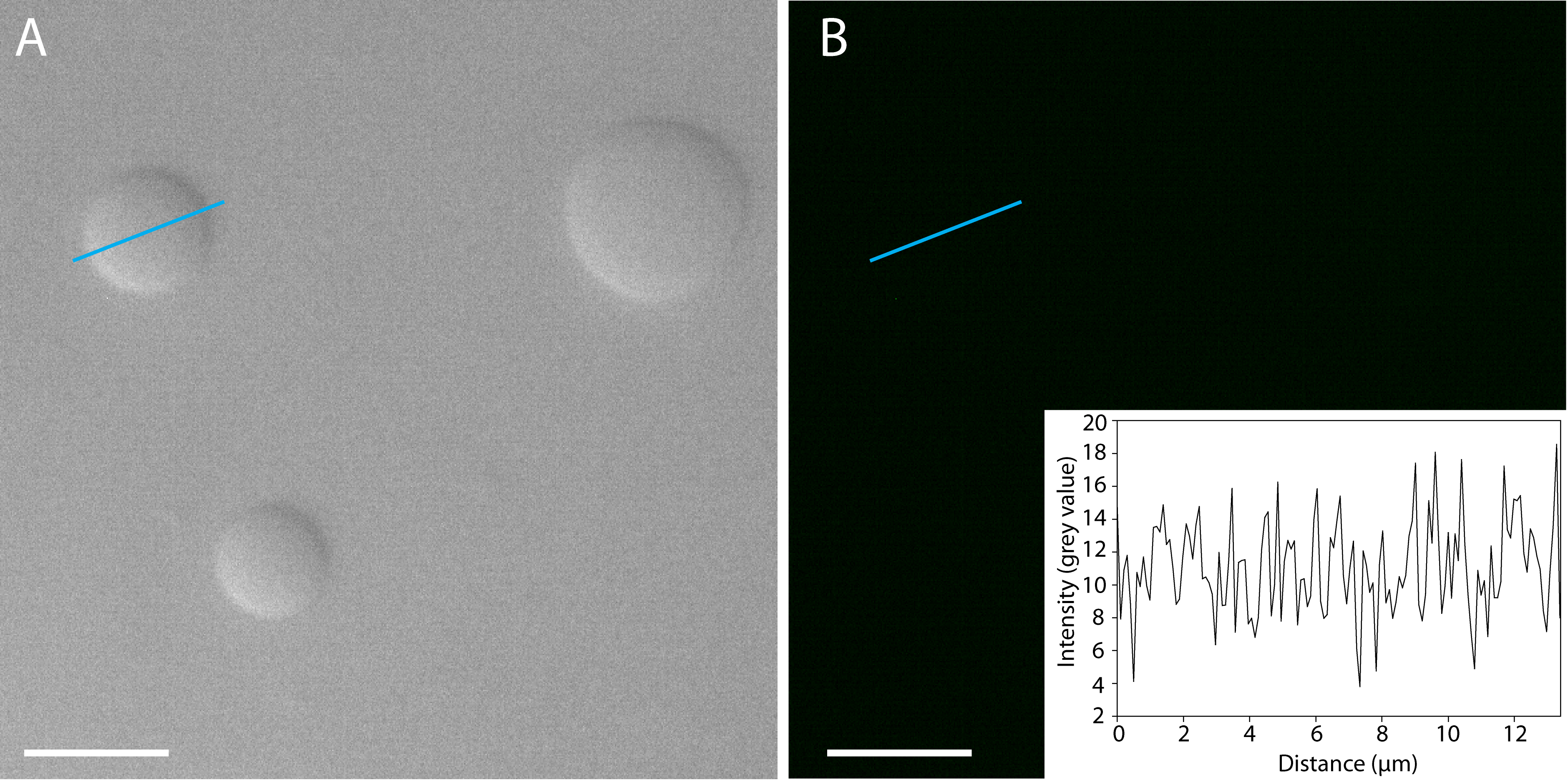

Supplement: Supplementary file 3 [file Image2.TIF]
